# Prognostic value of preoperative high-sensitivity C-reactive protein to albumin ratio in patients with dilated cardiomyopathy receiving pacemaker therapy: A retrospective two-center study in China

**DOI:** 10.1016/j.ijcha.2024.101554

**Published:** 2024-11-16

**Authors:** Jiaqi Pan, Enrui Zhang, Jie Han, Haiyu Zou, Liangrong Zheng

**Affiliations:** a Department of Cardiology, The First Affiliated Hospital, College of Medicine, Zhejiang University, Hangzhou, China; b Department of Cardiology, The First Affiliated Hospital, Nanjing Medical University, Nanjing, China; c Department of Cardiology, The Fourth Affiliated Hospital, Zhejiang University School of Medicine, Yiwu, Zhejiang, China

**Keywords:** high-sensitivity C-reactive protein to albumin ratio, Dilated cardiomyopathy, Cardiac resynchronization therapy, Implantable cardiac defibrillators, Major adverse cardiac events

## Abstract

**Background:**

Despite receiving pacemaker therapy, patients with heart failure with reduced ejection fraction (HFrEF) due to dilated cardiomyopathy (DCM) remain at an increased risk of adverse cardiovascular events. The high-sensitivity C-reactive protein (hs-CRP)-to-albumin ratio (CAR) is a novel indicator. This study aimed to assess the prognostic value of preoperative CAR in this population.

**Methods:**

Patients with DCM who underwent cardiac resynchronization therapy (CRT) or implantable cardiac defibrillator (ICD) implantation for HFrEF between 2018 and 2023 were involved. The primary endpoint was major adverse cardiac events (MACE). Cox regression models were used to investigate predictors for MACE. Receiver operating characteristic (ROC) curve analysis was utilized to evaluate the diagnostic efficacy and identify the optimal cutoff point.

**Results:**

We enrolled 250 patients, of whom 78 experienced MACE. Patients who experienced MACE had a significantly higher CAR than those without MACE (*p* < 0.001). Multivariate Cox regression analysis indicated CAR as an independent predictor for MACE (hazard ratio = 4.301, 95 % confidence interval [CI] 1.833–10.091, *p* < 0.001). ROC curve analysis demonstrated the discriminatory ability of CAR in predicting MACE (area under the curve [AUC] = 0.732, 95 % CI 0.666–0.792, *p* < 0.001), with an optimal threshold of 0.08. Furthermore, the incidence of MACE was significantly higher in the high-CAR (> 0.08) group compared to the low-CAR (≤ 0.08) group (48.8 % vs. 13.6 %, *p* < 0.001).

**Conclusion:**

Among patients with DCM and HFrEF treated with CRT or ICD, CAR can serve as an independent risk predictor, with higher levels associated with poorer outcomes.

## Graphical abstract

**Figure:**
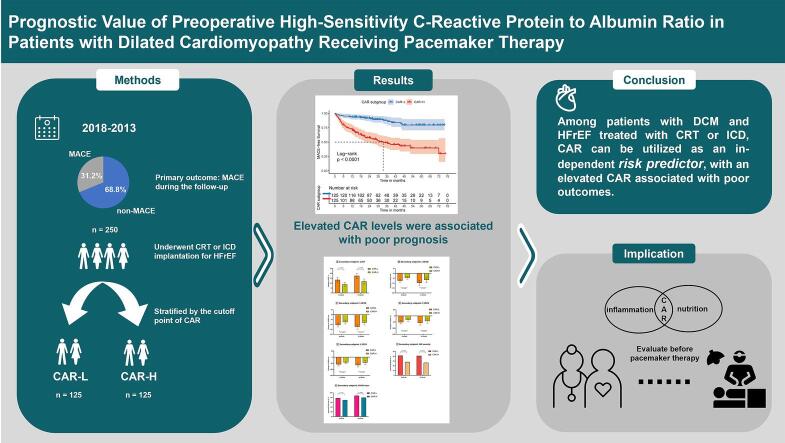

## 1 Introduction

Dilated cardiomyopathy (DCM) is a potentially life-threatening condition characterized by ventricular dilation and systolic dysfunction, occurring independently of coronary artery disease or abnormal loading conditions [1]. The annual prevalence of DCM is estimated to be 5 to 8 cases per 100,000 people [2]. DCM is one of the leading causes of diminished quality of life and cardiovascular mortality [3], and heart failure with reduced ejection fraction (HFrEF) is closely related to its progression [4], [5]. Adverse cardiac remodeling is a crucial indicator of poor prognosis [6]. Cardiac resynchronization therapy (CRT) and implantable cardiac defibrillators (ICD) have been widely utilized to reduce mortality, arrhythmic events, and hospitalizations in patients with DCM [1], [5]. However, adverse events persist, and not all patients benefit equally from these treatments. Therefore, it is essential to recognize patients who remain at high risk even after device implantation.

High-sensitivity C-reactive protein (hs-CRP) and serum albumin, both synthesized by hepatocytes, are independent predictors of poor outcomes in patients with DCM and heart failure [7], [8], [9], [10]. The hs-CRP to albumin ratio (CAR) provides a more comprehensive assessment of inflammatory and nutritional status than hs-CRP or albumin alone. As a novel inflammatory biomarker, CAR has become a valuable tool in clinical practice, particularly for prognostic assessments in various diseases, including chronic lymphocytic leukemia, sepsis, hepatocellular carcinoma, and coronary artery disease [11], [12], [13], [14]. Moreover, clinical evidence has linked CAR to HFrEF [15], [16]. While research has suggested that high CAR levels are associated with increased mortality risk in patients with ICD and HFrEF [15], the role of CAR in predicting prognosis among patients with DCM undergoing CRT or ICD implantation remains inconclusive.

Therefore, our primary objective was to assess whether preoperative CAR could be a novel indicator for predicting major adverse cardiac events (MACE) among patients with DCM receiving CRT or ICD for HFrEF. Furthermore, this study aimed to investigate the association between preoperative CAR and postoperative left ventricular reverse remodeling (LVRR), mitral regurgitation (MR), and cardiac function in this population.

## 2 Methods

### 2.1 Study design

**Fig. 1:**
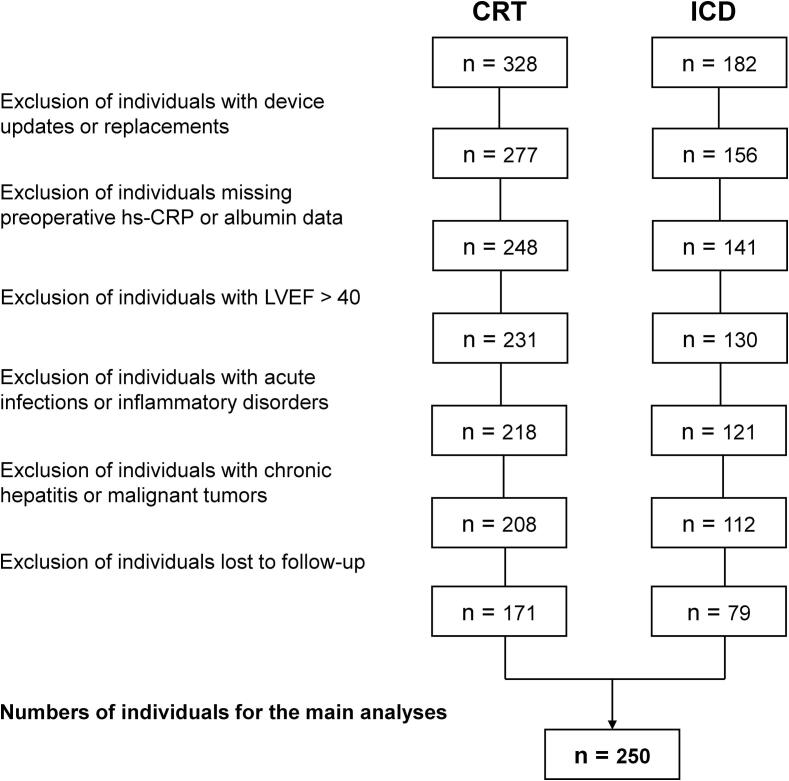
Flowchart demonstrating the design of our study. CRT, cardiac resynchronization therapy; hs-CRP, high-sensitivity C reactive protein; ICD, implantable cardioverter-defibrillator; LVEF, left ventricular ejection fraction.

This retrospective cohort study included consecutive patients with DCM who underwent CRT or ICD implantation for HFrEF. The study was carried out in the Department of Cardiology of the First Affiliated Hospital of Zhejiang University School of Medicine and the First Affiliated Hospital of Nanjing Medical University between January 2018 and April 2023. The diagnosis of DCM was confirmed through a comprehensive evaluation following European Society of Cardiology (ESC) Guidelines [17], including clinical and family history assessment, physical examination, laboratory measurements, electrocardiography, echocardiography, computed tomography coronary angiography, and coronary angiography. HFrEF was diagnosed based on the American Heart Association (AHA)/ American College of Cardiology (ACC)/ Heart Failure Society of America (HFSA) Guidelines [17]. The following criteria were applied for exclusion: (a) age < 18  years, (b) echocardiographic report showing LVEF > 40 % at screening, (c) loss to follow-up, (d) acute infections or inflammatory disorders before admission, (e) chronic hepatitis or malignant tumors before admission, and (f) missing values of hs-CRP or serum albumin concentration. The flowchart for our research design is presented in Fig. 1.

The study protocol conformed to the ethical guidelines of the 1975 Declaration of Helsinki and was approved by the Ethics Committee of the First Affiliated Hospital, Zhejiang University School of Medicine. Written informed consent was obtained from each participant.

### 2.2 Data collection and definition

Data on demographic characteristics, laboratory tests, electrocardiogram (ECG) findings, echocardiography parameters, discharge medications, and device types were extracted from the electronic database. In this study, CRT included left bundle branch pacing (LBBP) and biventricular pacing (BiVP). Diabetes mellitus (DM) was diagnosed based on the use of hypoglycemic medications or a fasting blood glucose level ≥ 7.0 mmol/L at admission. Atrial fibrillation was confirmed through ECG, dynamic ECG, or a self-reported history of atrial fibrillation attacks. The New York Heart Association (NYHA) functional class was assessed by experienced clinicians. ECG reports were analyzed and confirmed by specialized physicians. The severity of MR [18] and echocardiographic parameters, including left ventricular ejection fraction (LVEF), left ventricular end-diastolic diameter (LVEDD), left ventricular end-systolic diameter (LVESD), left ventricular end-diastolic volume (LVEDV), and left ventricular end-systolic volume (LVESV), were assessed using three-dimensional (3D) transthoracic echocardiography conducted by two experienced sonographers who were blinded to this study. Blood samples were collected in the morning after a 12-hour fast prior to device implantation. CAR (mg/g) was calculated by dividing hs-CRP levels (mg/L) by serum albumin concentration (g/L).

### 2.3 Study outcomes and follow-up

The primary outcome of our study was the occurrence of MACE during the follow-up period, which included all-cause mortality, re-hospitalization for heart failure, and ventricular arrhythmic events (sustained ventricular tachycardia and ventricular fibrillation requiring anti-tachycardia pacing, appropriate shock therapy, or pharmacological cardioversion). Secondary outcomes included changes in echocardiographic parameters (LVEF, LVEDD, LVESD, LVEDV, and LVESV), MR severity, and NYHA functional class from baseline to the 6-month and 12-month follow-ups. Moreover, the incidence of complications, including subcutaneous hematoma, cardiovascular implantable electronic device (CIED) pocket infection, lead dislodgement, pneumothorax, and device movement, was recorded and analyzed. All adverse events were reviewed and adjudicated centrally by two independent cardiologists, with disagreements resolved through consensus. The follow-up period in our study was defined as the duration from device implantation to the occurrence of MACE or the last censoring point for patients who survived without experiencing MACE. Follow-up data were collected from hospital records or telephone interviews conducted by well-trained investigators blinded to the study.

### 2.4 Statistical analysis

Quantitative data with a normal distribution were presented as the mean ± standard deviation, while non-normally distributed data were expressed as the median (interquartile range, IQR). Categorical variables were represented as numbers and percentages. To evaluate the normality of the data, the Kolmogorov–Smirnov test was employed. Differences among subgroups for quantitative variables were assessed using either the student's *t*-test or the Mann–Whitney *U* test, as appropriate, and categorical data were analyzed using the Pearson χ2 test or Fisher's exact test. Correlation between hs-CRP and albumin, as well as collinearity diagnostics, were performed using linear regression analysis. The univariate Cox proportional hazards model was used to identify significant variables linked to the prediction of MACE. After eliminating variables with significant collinearity, those with *p* < 0.05 in the univariate Cox regression analysis were further included in the multivariate analysis. Receiver operating characteristic (ROC) curves were constructed to determine the predictive capabilities of CAR and other parameters for MACE. Delong's test was used to compare the area under the ROC curve (AUC). The optimal cutoff point of CAR was identified using Youden's index (sensitivity + specificity − 1) to achieve the highest sensitivity and specificity. Patients with CAR above this cutoff point were classified into the high-CAR (CAR-H) group, while those with CAR below or equal to the cutoff point were classified into the low-CAR (CAR-L) group. Survival curves were generated using the Kaplan–Meier method and compared with the log-rank testing. All statistical analyses were two-sided, with a *p*-value of less than 0.05 considered statistically significant.

Statistical analyses were conducted using SPSS version 26.0 (IBM, Chicago, IL, USA), while GraphPad Prism 10 and R software 4.2.2 were used to create figures. The R packages “survival” and “survminer” were used for survival analysis.

## 3 Results

### 3.1 Baseline characteristics of patients

Following the inclusion and exclusion criteria, our study enrolled 250 patients with HFrEF and DCM. Of these, 171 (68.4 %) received CRT, while 79 (31.6 %) received ICD. The median age at the time of operation was 64.0 years [IQR, 57.0–71.0] years, and 65.2 % were men. Over a median follow-up duration of 26.0 (14.0–41.4) months, 78 (31.2 %) patients experienced MACE, which included 8 cases of all-cause mortality, 52 cases of re-hospitalization for heart failure, and 18 cases of ventricular arrhythmias. Patients who experienced MACE were classified as the MACE group, while those without MACE were classified as the non-MACE group.

Comparative analysis revealed that patients in the MACE group were older (69.0 years [57.0–74.0] vs. 64.0 [56.0–70.0], *p* = 0.008) and had elevated levels of hs-CRP, serum creatinine, Troponin I, and N-terminal pro-brain natriuretic peptide (NT-proBNP). Conversely, they had lower serum albumin concentration, estimated glomerular filtration rate (eGFR), and hemoglobin. Echocardiographic assessments indicated that the MACE group had larger LVEDD, LVESD, LVEDV, and LVESV compared to the non-MACE group. Furthermore, the MACE group demonstrated a significantly higher CAR (0.16 [0.08–0.30] vs. 0.05 [0.02–0.14], *p* < 0.001). Detailed demographic data, laboratory test results, NYHA classification, electrocardiogram (ECG) findings, MR severity, echocardiographic parameters, discharge medications, and device types are summarized in Table 1.

**Table 1:** Baseline characteristics of patients in the MACE and non-MACE groups.

	Total (n = 250)	non-MACE group (n = 172)	MACE group (n = 78)	*P* value
Demographics				
Age, y	64.0 (57.0–71.0)	64.0 (56.0–70.0)	69.0 (57.0–74.0)	0.008*
Male gender	163 (65.2)	107 (62.2)	56 (71.8)	0.140
Diabetes mellitus	42 (16.8)	25 (14.5)	17 (21.8)	0.155
Atrial fibrillation	51 (20.4)	30 (17.4)	21 (26.9)	0.085
Statin uptake	102 (40.8)	70 (40.7)	32 (41.0)	0.961
Laboratory values				
Serum albumin, g/L	39.1 ± 4.4	40.1 ± 4.1	37.0 ± 4.3	＜0.001*
Hs-CRP, mg/L	3.10 (0.90–7.23)	1.85 (0.71–5.24)	5.87 (3.18–10.28)	＜0.001*
**CAR, mg/g**	**0.08 (0.02**–**0.18)**	**0.05 (0.02**–**0.14)**	**0.16 (0.08**–**0.30)**	＜0.001*
Serum creatinine, μmol/L	86.0 (73.0–106.3)	83.5 (71.1–100.8)	92.5 (78.0–128.4)	0.001*
eGFR, mL/min	74.3 (54.9–88.1)	76.4 (63.1–90.0)	68.5 (44.5–84.1)	0.003*
Hemoglobin, g/L	136.8 ± 17.7	138.8 ± 16.2	132.5 ± 19.8	0.009*
D-dimer, μg/L FEU	430.5 (210.8–1102.5)	413.0 (180.0–1050.0)	468.0 (256.0–1229.0)	0.280
Troponin I, ng/mL	0.027 (0.012–0.053)	0.023 (0.011–0.045)	0.035 (0.018–0.080)	0.002*
Lactate dehydrogenase, U/L	209.0 (172.5–252.0)	207.0 (172.0–250.0)	221.0 (172.8–257.3)	0.261
CK-MB, U/L	15.0 (12.0–21.3)	15.0 (12.0–22.0)	14.0 (12.0–21.0)	0.647
LDL-C, mmol/L	2.14 (1.70–2.73)	2.17 (1.70–2.76)	2.08 (1.72–2.39)	0.345
Serum potassium, mmol/L	4.11 (3.78–4.40)	4.10 (3.77–4.35)	4.12 (3.86–4.48)	0.500
NT-proBNP, pg/mL	1011.0 (298.2–3109.0)	721.0 (246.5–2507.0)	1740.0 (443.8–4165.8)	0.003*
NYHA classification				0.155
II	49 (19.6)	36 (20.9)	13 (16.7)	
III	143 (57.2)	102 (59.3)	41 (52.6)	
IV	58 (23.2)	34 (19.8)	24 (30.8)	
ECG findings				
QRS duration, ms	151.0 (110.0–170.0)	151.0 (110.0–170.0)	148.5 (110.0–168.5)	0.778
LBBB	134 (53.6)	99 (57.6)	35 (44.9)	0.062
MR severity				0.484
Mild	94 (37.6)	67 (39.0)	27 (34.6)	
Moderate	140 (56.0)	96 (55.8)	44 (56.4)	
Severe	16 (6.4)	9 (5.2)	7 (9.0)	
Echocardiography parameters				
LVEF, %	29.0 (25.0–34.0)	30.0 (25.0–34.0)	28.0 (25.0–32.3)	0.118
LVEDD, mm	65.9 (61.0–72.9)	64.6 (60.7–72.0)	69.4 (61.7–77.0)	0.007*
LVESD, mm	57.5 (51.2–64.6)	56.9 (50.2–63.2)	60.3 (52.0–68.2)	0.011*
LVEDV, mL	220.5 (184.0–284.2)	212.0 (183.0–271.5)	254.6 (196.8–320.0)	0.006*
LVESV, mL	161.8 (126.0–213.7)	156.6 (123.0–205.8)	184.3 (128.0–241.6)	0.016*
Medications at discharge				
Beta-blockers	216 (86.4)	150 (87.2)	66 (84.6)	0.579
ACEIs/ARBs/ARNI	197 (78.8)	141 (82.0)	56 (71.8)	0.068
SGLT-2i	50 (20.0)	38 (22.1)	12 (24.0)	0.219
MRAs	213 (85.2)	148 (86.0)	65 (83.3)	0.576
Diuretics	210 (84.0)	144 (83.7)	66 (84.6)	0.858
Types				0.116
CRT	171 (68.4)	123 (71.5)	48 (61.5)	
ICD	79 (31.6)	49 (28.5)	30 (38.5)	

Values were presented as mean ± SD, median (interquartile range) or n (%). *p value < 0.05.

Abbreviations: ACEIs, Angiotensin-converting enzyme inhibitors; ARBs, Angiotensin II receptor blockers; ARNI, Angiotensin Receptor-Neprilysin Inhibitor; CAR, high-sensitivity C-reactive protein to albumin ratio; CK-MB, creatine kinase isoenzymes; CRT, cardiac re-synchronization therapy; eGFR, estimated glomerular filtration rate; ECG, electrocardiogram; Hs-CRP, high-sensitivity C-reactive protein; ICD, implantable cardioverter-defibrillator; LDL-C, low-density lipoprotein cholesterol; LBBB, left bundle branch block; LVEF, left ventricular ejection fraction; LVEDD, left ventricular end-diastolic diameter; LVESD, left ventricular end-systolic diameter; LVEDV, left-ventricular end-diastolic volume; LVESV, left ventricular end-systolic volume; MACE, major adverse cardiovascular events; MR, mitral regurgitation; MRA, mineralocorticoid receptor antagonists; NT-pro-BNP, N-terminal pro-brain natriuretic peptide; NYHA, New York heart as-sociation; SGLT-2i Sodium-Glucose Transport Protein 2 Inhibitors.

### 3.2 Independent predictors for MACE

The independent predictors for MACE identified through univariate and multivariate Cox regression models are presented in Table 2. The results of the univariate Cox regression analysis indicated that age, atrial fibrillation, hs-CRP, serum albumin, CAR, serum creatinine, eGFR, hemoglobin, lactate dehydrogenase (LDH), log-transformed NT-pro BNP, LVEDD, LVESD, LVEDV, and LVESV were significantly associated with the risk of MACE. Variables with *p* < 0.05 in the univariate analysis were incorporated into the multivariate Cox regression model. The results demonstrated that eGFR (hazard ratio [HR] = 0.987, 95 % confidence interval [CI] 0.975–0.999, *p* = 0.039), LVESV (HR = 1.005, 95 % CI 1.002–1.008, *p* = 0.001), and CAR (HR = 4.072, 95 % CI 1.796–9.234, *p* = 0.001) were independent predictors for MACE. Specifically, each unit increase in CAR (mg/L) was associated with a 3.072-fold increase in the risk of MACE. Similarly, each unit increase in LVESV (mL) corresponded to a 0.5 % increase in MACE risk, whereas every unit increase in eGFR (mL/min) was linked to a 1.3 % decrease in MACE risk. Notably, no significant difference in MACE occurrence was observed between the two types of devices.

**Table 2:** Univariate and multivariate analysis for MACE.

Variables	Univariate analysis			Multivariate analysis	
	HR (95 % Cl)	*P* value		HR (95 % Cl)	*P* value
Age, y	1.031 (1.008–1.054)	0.009*		1.013 (0.987–1.040)	0.327
Gender					
Female	Reference				
Male	1.495 (0.913–2.449)	0.110			
Diabetes mellitus	1.549 (0.904–2.654)	0.111			
Atrial fibrillation	1.742 (1.055–2.875)	0.030*		1.460 (0.813–2.625)	0.205
Statin uptake	1.022 (0.651–1.606)	0.923			
Hs-CRP, mg/L	1.050 (1.030–1.071)	<0.001*			
Serum albumin, g/L	0.864 (0.818–0.913)	<0.001*			
**CAR, mg/g**	**6.337 (3.198**–**12.558)**	<0.001*		**4.072 (1.796**–**9.234)**	0.001*
Serum creatinine, μmol/L	1.002 (1.001–1.003)	0.005*			
eGFR, mL/min	0.980 (0.971–0.990)	<0.001*		0.987 (0.975–0.999)	0.039*
Hemoglobin, g/L	0.983 (0.970–0.997)	0.017*		0.993 (0.978–1.008)	0.364
Log D-dimer, μg/L FEU	1.297 (0.785–2.142)	0.310			
Troponin I, ng/mL	1.035 (0.625–1.714)	0.894			
Lactate dehydrogenase, U/L	1.003 (1.000–1.006)	0.036*		1.002 (0.999–1.005)	0.268
CK-MB, U/L	1.000 (0.974–1.028)	0.974			
LDL-C, mmol/L	0.830 (0.599–1.151)	0.264			
Serum potassium, mmol/L	1.287 (0.841–1.971)	0.245			
Log NT-proBNP, pg/mL	1.699 (1.183–2.441)	0.004*		0.980 (0.631–1.523)	0.929
NYHA class					
II	Reference				
III	1.112 (0.595–2.076)	0.739			
IV	1.775 (0.903–3.489)	0.511			
MR severity					
Mild					
Moderate	1.063 (0.658–1.718)	0.802			
Severe	1.909 (0.828–4.400)	0.129			
QRS duration, ms	0.999 (0.992–1.007)	0.856			
LBBB	0.651 (0.417–1.018)	0.060			
LVEF, %	0.965 (0.928–1.003)	0.074			
LVEDD, mm	1.037 (1.013–1.061)	0.002*			
LVESD, mm	1.031 (1.009–1.054)	0.006*			
LVEDV, mL	1.004 (1.002–1.007)	0.001*			
LVESV, mL	1.004 (1.001–1.007)	0.005*		1.005 (1.002–1.008)	0.001*
Beta-blockers	0.871 (0.471–1.614)	0.662			
ACEIs/ARBs/ARNI	0.689 (0.419–1.134)	0.143			
SGLT-2i	1.095 (0.581–2.061)	0.780			
MRAs	0.865 (0.477–1.569）	0.633			
Diuretics	1.089 (0.588–2.015)	0.787			
Types					
ICD	Reference				
CRT	0.651 (0.413–1.029)	0.066			

Abbreviations: ACEIs, Angiotensin-converting enzyme inhibitors; ARBs, Angiotensin II receptor blockers; ARNI, Angiotensin Receptor-Neprilysin Inhibitor; CI, confidence interval; CAR, high-sensitivity C-reactive protein to albumin ratio; CK-MB, creatine kinase isoenzymes; CRT, cardiac resynchronization therapy; eGFR, estimated glomerular filtration rate; HR, hazard ratio; Hs-CRP, high-sensitivity C-reactive protein; ICD, implantable cardioverter-defibrillator; LDL-C, low-density lipoprotein cholesterol; LBBB, left bundle branch block; LVEF, left ventricular ejection fraction; LVEDD, left ventricular end diastolic-diameter; LVESD, left ventricular end-systolic diameter; LVEDV, left ventricular end diastolic-volume; LVESV, left ventricular end-systolic volume; MACE, major adverse cardiovascular events; MR, mitral regurgitation; MRA, mineralocorticoid receptor antagonists; NT-pro-BNP, N-terminal pro-brain natriuretic peptide; NYHA, New York heart association; RBBB, right bundle branch block; SGLT-2i Sodium-Glucose Transport Protein 2 Inhibitors.

*p value < 0.05.

### 3.3 Diagnostic efficacy of CAR

**Fig. 2:**
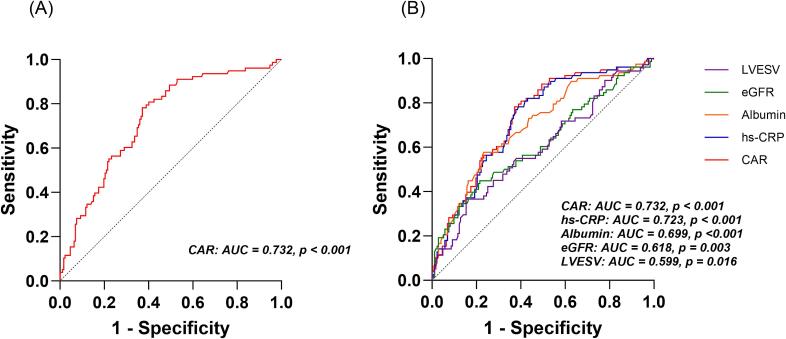
ROC curve analyses for assessing the predictive value of (A) CAR; (B) CAR and other risk valuables. CAR, high-sensitivity C-reactive protein to albumin ratio; eGFR, estimated glomerular filtration rate; hs-CRP, high-sensitivity C-reactive protein; LVESV, left ventricular end-systolic volume; ROC, Receiver operating characteristic.

According to the multivariate Cox regression outcomes, ROC curve analyses were conducted to assess the predictive performance of CAR and other risk factors for MACE. As shown in Sup. Table 1, the AUC for CAR in predicting MACE was 0.732 (95 % CI 0.666–0.792, *p* < 0.001). The optimal cutoff point of CAR was 0.08, with a sensitivity of 0.782 and a specificity of 0.628. The AUC for hs-CRP, albumin, eGFR, and LVESV was 0.723 (95 % CI 0.657–0.789, *p* < 0.001), 0.699 (95 % CI 0.629–0.770, *p* < 0.001), 0.618 (95 % CI 0.540–0.697, *p* = 0.003), and 0.599 (95 % CI 0.519–0.680, *p* = 0.016), respectively. These values were lower than that of CAR (*p* < 0.05). Based on the ROC curves, CAR demonstrated greater predictive efficacy for MACE compared to hs-CRP, albumin, eGFR, and LVESV (Fig. 2).

### 3.4 Correlation between hs‑CRP and albumin

Linear regression analysis explored the correlation between hs-CRP and albumin levels. The hs-CRP levels ranged from 0.07 to 45.32 mg/L, and the albumin concentrations ranged from 28.30 to 49.80  g/L. The correlation analysis indicated a negative relationship between hs-CRP and albumin levels (R^2^ = 0.089, Standard β = − 0.172, *p* < 0.001) (Supplementary Fig. 1).

### 3.5 Comparison of improvement in MR and LVEF

The improvement in MR, including reductions in severity from severe to moderate, severe to mild, and moderate to mild, was significantly greater in the non-MACE group compared to the MACE group at both the 6-month (75/172 [43.6 %] vs. 12/78 [15.4 %], *p* < 0.001) and 12-month follow-ups (70/172 [40.7 %] vs. 14/78 [17.9 %], *p* < 0.001). Similarly, the improvement in LVEF was significantly greater in the non-MACE group compared to the MACE group at both 6 months (13.6 [95 % CI 11.6–15.5] vs. 4.8 [95 % CI 2.8–6.9], *p* < 0.001) and 12 months (17.8 [95 % CI 15.8–19.9] vs. 6.5 [95 % CI 3.5–9.4], *p* < 0.001).

### 3.6 CAR and clinical outcomes

**Fig. 3:**
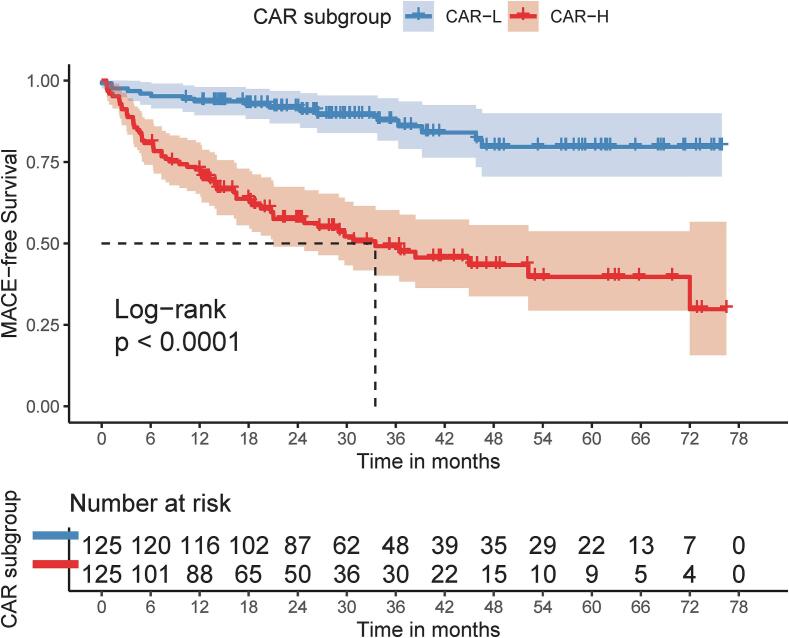
Kaplan-Meier curves for primary outcome stratified by the cutoff point of CAR. CAR, high-sensitivity C-reactive protein to albumin ratio; MACE, major adverse cardiovascular events.

Our results demonstrated that patients in the MACE group exhibited higher CAR levels than those in the non-MACE group (0.16 [0.08–0.30] vs. 0.05 [0.02–0.14], *p* < 0.001) (Supplementary Fig. 2A). Based on the cutoff point of CAR, the study population was divided into two groups: CAR-L (CAR ≤ 0.08) and CAR-H (CAR > 0.08). The cumulative incidence of MACE was significantly higher in the CAR-H group compared to the CAR-L group (61/125 [48.8 %] vs. 17/125 [13.6 %], *p* < 0.001) (Table 3, Supplementary Fig. 2B). The Kaplan–Meier curves (Fig. 3) indicated that patients with higher CAR levels had a lower MACE-free survival rate compared to those with lower CAR levels in patients with DCM who underwent CRT or ICD implantation.

**Table 3:** Primary and Secondary outcomes between CAR groups.

	CAR-L	CAR −H	*P* value
Primary outcome			
MACE	17 (13.6)	61 (48.8)	＜0.001*
Secondary outcomes			
Echocardiography variables			
Change in LVEF from baseline to 6 mo, %	13.3 (11.0, 15.7)	8.8 (6.7, 10.9)	0.005*
Change in LVEF from baseline to 12 mo, %	17.5 (14.9, 20.1)	11.7 (9.1, 14.3)	0.002*
Change in LVEDD from baseline to 6 mo, mm	−7.5 (−9.2, −5.9)	−4.5 (−6.1, −3.1)	0.009*
Change in LVEDD from baseline to 12 mo, mm	−9.7 (−12.2, −7.2)	−6.7 (−9.5, −3.9)	0.112
Change in LVESD from baseline to 6 mo, mm	−10.5 (−12.5, −8.6)	−7.5 (−9.5, −5.5)	0.032*
Change in LVESD from baseline to 12 mo, mm	−12.4 (−14.9, −9.8)	−8.1 (−10.6, −5.6)	0.017*
Change in LVEDV from baseline to 6 mo, mL	−51.5 (−65.3, −37.7)	–32.4 (−44.1, −20.7)	0.037*
Change in LVEDV from baseline to 12 mo, mL	−57.4 (−75.2, −39.6)	−41.7 (−59.7, –23.7)	0.218
Change in LVESV from baseline to 6 mo, mL	−58.6 (−71.9, −45.2)	−43.0 (−54.0, –32.1)	0.074
Change in LVESV from baseline to 12 mo, mL	−64.4 (−80.1, −48.8)	−44.0 (−60.9, −27.0)	0.078
MR severity			
Improve MR grade ≥ 1 from baseline to 6 mo	52 (41.6)	35 (28.0)	0.024*
Improve MR grade ≥ 1 from baseline to 12 mo	51 (40.8)	33 (26.4)	0.016*
Cardiac function			
Improve NYHA class ≥ 1 from baseline to 6 mo	97 (77.6)	87 (69.6)	0.151
Improve NYHA class ≥ 1 from baseline to 12 mo	110 (88.0)	100 (80.0)	0.084

Values were presented as mean (95 % CI) or n (%). *p value < 0.05.

Abbreviations: CAR, high-sensitivity C reactive protein to albumin ratio; LVEF, left ventricular ejection fraction; LVEDD, left ventricular end diastolic diameter; LVESD, left ventricular end systolic diameter; LVEDV, left ventricular end diastolic volume; LVESV, left ventricular end systolic volume; MACE, major adverse cardiovascular events; MR, mitral regurgitation; NYHA, New York heart association.

**Fig. 4:**
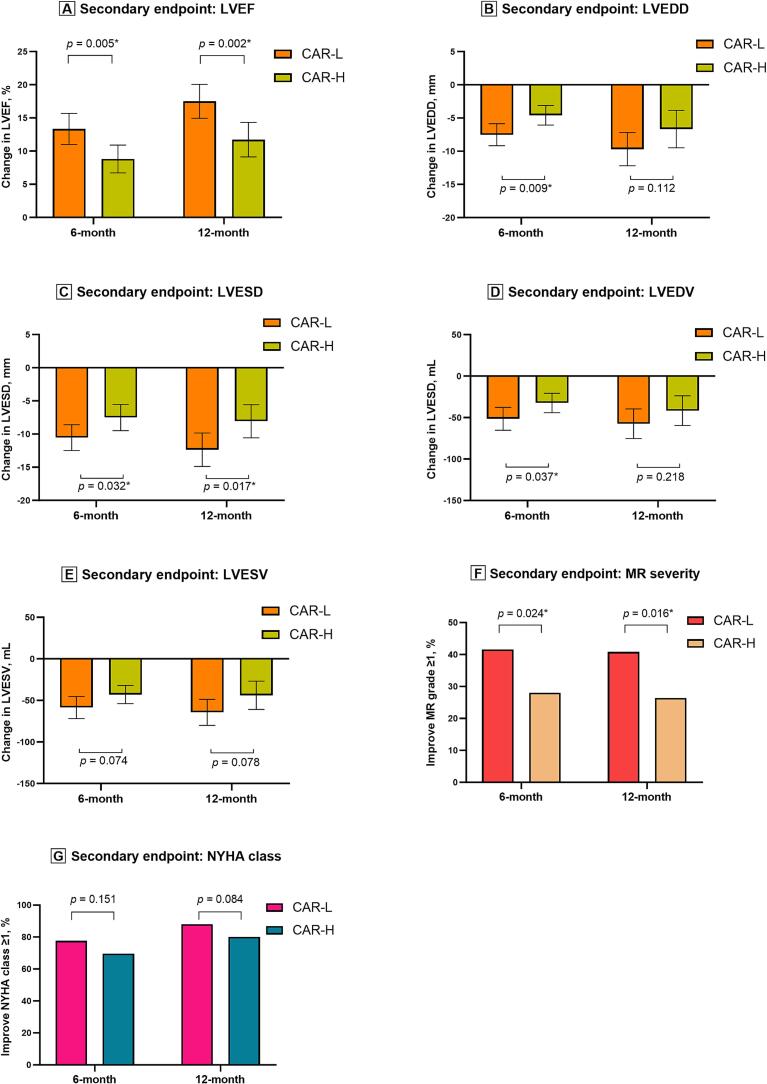
Changes in LVEF (A), LVEDD (B), LVESD (C), LVEDV (D), LVESV (E), MR severity (F), and NYHA class (G) from baseline to the 6-month and 12-month follow-ups. LVEF, left ventricular ejection fraction; LVEDD, left ventricular end-diastolic diameter; LVESD, left ventricular end-systolic diameter; LVEDV, left-ventricular end-diastolic volume; LVESV, left ventricular end-systolic volume; MR, mitral regurgitation; NYHA, New York heart association. *p value < 0.05.

Changes in LVEF, LVEDD, LVESD, LVEDV, and LVESV from baseline to the 6-month and 12-month follow-ups were compared between the CAR-L and CAR-H groups (Table 3). After 6 months, the CAR-L group showed a more significant increase in LVEF and more substantial decreases in LVEDD, LVESD, and LVEDV compared to the CAR-H group. After 12 months, the increase in LVEF and the reduction in LVESD remained more pronounced in the CAR-L group (Fig. 4). These findings suggest that high CAR levels adversely affect LVRR, especially in the early stages following device implantation. Analogously, the improvement in MR was significantly greater in the CAR-L group compared to the CAR-H group at both the 6-month and 12-month follow-ups (Table 3, Fig. 4). However, the improvement in the NYHA class was comparable between the CAR-L and CAR-H groups after the 6-month and 12-month follow-ups (Table 3, Fig. 4).

The difference in surgical complications between the CAR-L and CAR-H groups was also analyzed (Sup. Table 2). Complications occurred in 35 patients (14.0 %), including 5 cases of subcutaneous hematoma, 3 of CIED pocket infection, 3 of lead dislodgement, and 2 of device movement in the CAR-L group. In the CAR-H group, there were 8 cases of subcutaneous hematoma, 8 of CIED pocket infection, 4 of lead dislodgement, and 2 of pneumothorax. All patients with complications received appropriate treatment and recovered during the follow-up. There was no significant difference in the overall incidence of complications between the two groups (13 in the CAR-L group [10.4 %] vs. 22 in the CAR-H group [17.6 %], *p* = 0.101).

## 4 Discussion

Our study is the first to demonstrate that preoperative CAR can serve as a novel biomarker for predicting prognosis in patients with DCM receiving CRT or ICD for HFrEF.

DCM is a severe cardiac condition characterized by structural or functional defects in the heart muscle, resulting in complications including heart failure and malignant arrhythmias, which significantly diminish the quality of life [1]. The 2023 ESC Guidelines recommend CRT and ICD for patients with DCM and HFrEF to reduce morbidity and mortality [19]. Despite optimal device therapy, many patients continue to experience poor outcomes. Identification of high-risk patients before device implantation remains challenging. Our study suggests that CAR can act as an independent prognostic indicator to address this issue.

Decades of research have established inflammation and nutritional deficiency as contributors to DCM [20]. Hs-CRP, an acute-phase protein produced by liver cells in response to inflammatory stimuli, is highly correlated with cardiovascular disease (CVD) risk in the general population [21]. Levels of hs-CRP > 3 mg/L frequently indicate high cardiovascular risks [22]. Moreover, hs-CRP plays a crucial role in the progression of ventricular remodeling, characterized by interstitial myocardial fibrosis [23]. It has been shown to be an independent predictor of prognosis in patients with DCM [8]. Albumin, a key nutritional indicator, is also closely connected to the inflammatory response [24]. Inflammation may reduce albumin synthesis and increase its degradation through pro-inflammatory cytokines, including interleukin-6 (IL-6), IL-1β, and tumor necrotic factor-alpha (TNF-α) [25]. Hypoalbuminemia is independently associated with worse outcomes in several CVDs, such as heart failure [26]. A recent study involving 1058 older patients with DCM found that hypoalbuminemia was associated with a higher risk of long-term mortality [9]. Consistent with previous studies, our univariate Cox regression analysis indicated that elevated levels of hs-CRP and low albumin concentrations were associated with a higher probability of MACE in patients with DCM who underwent CRT or ICD implantation. Moreover, our linear regression analysis revealed a negative correlation between hs-CRP and albumin in this population.

Compared to hs-CRP or albumin alone, CAR provides a more comprehensive evaluation of the inflammatory condition and nutritional status. Several observational cohort studies have demonstrated the prognostic capability of CAR in CVD: Karabağ et al*.* [27] demonstrated that CAR could predict angiographic No-reflow in patients with ST-elevation myocardial infarction who underwent primary percutaneous coronary intervention and had a better predictive value than other systemic inflammatory markers, such as neutrophil-to-lymphocyte ratio. Similarly, Li et al*.* [28] found that in patients with type 2 diabetes mellitus undergoing percutaneous coronary intervention, an elevated CAR was linked to an increased risk of 5-year all-cause mortality and cardiac mortality. Seoudy et al*.* [29] also reported that high CAR levels in patients who received transcatheter aortic valve replacement were associated with a higher probability of all-cause mortality. In addition, Ozkan et al*.* [30] identified CAR as an independent predictor for the recurrence of atrial fibrillation after cryoablation.

The above studies highlight the predictive role of CAR in patients with CVD undergoing cardiac intervention. However, the prognostic efficacy of CAR in patients with CIED implantations remains underexplored. In this study, high CAR levels were strongly associated with an increased risk of MACE in patients with DCM who received CRT or ICD. The CAR level in the MACE group was significantly higher than that in the non-MACE group (*p* < 0.001), with the incidence of MACE rising considerably from 13.6 % in the CAR-L group to 48.8 % in the CAR-H (*p* < 0.001). Both univariate and multivariate Cox regression analyses confirmed the predictive value of CAR. Additionally, baseline eGFR and LVESV were identified as risk predictors for MACE, consistent with previous research [31], [32]. ROC curve analysis indicated that CAR exhibited a higher AUC value than hs-CRP, albumin, eGFR, and LVESV, suggesting greater discriminatory power for predicting MACE.

Another crucial aspect of our study was the exploration of the link between CAR, LVRR, MR severity, and cardiac function changes. LVRR is a favorable response in patients with DCM, characterized by improved LVEF and reduced left ventricular dimensions and volumes [33], [34]. Inflammation and malnutrition contribute to the progression of left ventricular remodeling [23], [35], while CIED, such as CRT, enhances LVRR, particularly in patients with mechanical dyssynchrony [4]. Antonio et al. reported that reduced hs-CRP levels correlated with LVRR in patients with HFrEF who underwent CRT [36]. Belén et al. demonstrated that pre-CRT nutritional status was linked to LVRR [37]. Our study showed that an elevated CAR adversely affected LVRR, especially in an early postoperative stage (6 months). These findings lead to the hypothesis that CAR levels are closely associated with LVRR in patients with DCM and CIED. MR has been shown to be a common complication in CRT patients with DCM and has been linked to LVRR [38]. Our study further indicated an association between preoperative CAR levels and postoperative MR severity following CIED implantation. While most patients with DCM experienced significant improvements in cardiac function at 6 and 12 months postoperatively, no significant differences were observed between the CAR-L and CAR-H groups.

This study had several clinical implications. As a novel indicator, CAR is inexpensive, accessible, reproducible, and practical, allowing for the early identification of patients at high risk. Our findings demonstrated that elevated preoperative CAR levels are strongly associated with poor prognosis among patients with DCM receiving CRT or ICD for HFrEF, underscoring the importance of physicians improving patients’ inflammatory and nutritional status before CIED implantation. After CIED implantation, we humbly suggest that patients with high preoperative CAR levels require stricter heart failure medication management and undergo more frequent follow-ups to assess cardiac function and monitor pacemaker performance compared to those with low preoperative CAR levels. A multidisciplinary approach involving cardiologists, nutritionists, and immunologists could also benefit patients with elevated CAR levels. Furthermore, our results support the potential use of CAR as an assessment tool for LVRR in patients with DCM and CIED.

Several limitations must be acknowledged in our study. First, as a retrospective study, the ability to infer causality and generalizability is limited. Second, only a single preoperative CAR measurement was assessed, rather than a series of measurements, which could have provided more valuable insights into treatment responses. Third, it was impossible to fully adjust for all confounding factors associated with outcome risks, and we did not compare CAR with other novel inflammation markers, such as neutrophil-to-lymphocyte ratio, platelet-to-lymphocyte ratio, and HALP score [39]. Fourth, although most patients adhered to their follow-up visits and medication regimens, a small number did not, potentially impacting our clinical outcomes [40]. Lastly, the relatively small number of DCM patients treated with ICD implantation limited the generalizability of our findings to a broader population. Therefore, prospective and large-scale studies are needed to further explore CAR’s roles in identifying high-risk patients after device implantation.

## 5 Conclusion

This real-world study demonstrated that preoperative CAR is an effective and accessible indicator for predicting prognosis in patients with DCM undergoing CRT or ICD implantation. Elevated CAR levels were associated with an increased risk of MACE and adverse effects on LVRR. These findings suggest that preoperative inflammatory biomarkers and nutritional status should receive more attention during the preoperative evaluation. However, further prospective and large-scale studies are necessary to validate these findings.

## Declaration of generative AI and AI-assisted technologies in the writing process

During the preparation of this work the author Jiaqi Pan used ChatGPT in order to polish language. After using this tool, the author reviewed and edited the content as needed and takes full responsibility for the content of the publication.

## CRediT authorship contribution statement

**Jiaqi Pan:** Writing – original draft, Supervision, Methodology, Investigation, Formal analysis, Data curation, Conceptualization. **Enrui Zhang:** Investigation, Data curation. **Jie Han:** Funding acquisition. **Haiyu Zou:** Formal analysis. **Liangrong Zheng:** Writing – review & editing, Resources, Project administration, Conceptualization.

## Declaration of competing interest

The authors declare that they have no known competing financial interests or personal relationships that could have appeared to influence the work reported in this paper.
