# Supplementary material for: Prognostic value of preoperative high-sensitivity C-reactive protein to albumin ratio in patients with dilated cardiomyopathy receiving pacemaker therapy: A retrospective two-center study in China
Source: Int J Cardiol Heart Vasc. 2024 Nov 16;55:101554. doi: 10.1016/j.ijcha.2024.101554 (PMC11612793; doi:10.1016/j.ijcha.2024.101554)
Supplement: Supplementary Data 1 [file mmc1.docx]

| Parameters | AUC | Cut off points | Sensitivity | Specificity | 95% CI | *p* value |
| --- | --- | --- | --- | --- | --- | --- |
| CAR | 0.732 | 0.08 | 0.782 | 0.628 | 0.666-0.792 | <0.001 |
| Hs-CRP | 0.723 | 3.05 | 0.782 | 0.610 | 0.657-0.789 | <0.001 |
| Albumin | 0.699 | 37.2 | 0.577 | 0.767 | 0.629-0.770 | <0.001 |
| eGFR | 0.618 | 60.7 | 0.449 | 0.785 | 0.540-0.697 | 0.003 |
| LVESV | 0.599 | 221.0 | 0.366 | 0.847 | 0.519-0.680 | 0.016 |

Supplementary Table 1. Predictive ability of CAR and other risk factors in predicting MACE

Abbreviations: CAR, high-sensitivity C-reactive protein to albumin ratio; eGFR, estimated glomerular filtration rate; Hs-CRP, high-sensitivity C-reactive protein; LVESV, left ventricular end-systolic volume; MACE, major adverse cardiovascular events.

Supplementary Table 2. Complications of the study population

| Complications | CAR-L (n=125) | CAR-H (n = 125) | Total (n = 250) |
| --- | --- | --- | --- |
| All, n (%) | 13 (10.4) | 22 (17.6) | 35 (14.0) |
| Subcutaneous hematoma, n (%) | 5 (4.0) | 8 (6.4) | 13 (5.2) |
| CIED pocket infection, n (%) | 3 (2.4) | 8 (6.4) | 11 (4.4) |
| Lead dislodgement, n (%) | 3 (2.4) | 4 (3.2) | 7 (2.8) |
| Pneumothorax, n (%) | 0 (0.0) | 2 (1.6) | 2 (0.8) |
| Device movement, n (%) | 2 (1.6) | 0 (0.0) | 2 (0.8) |

CAR, high-sensitivity C reactive protein to albumin ratio; CIED, Cardiovascular Implantable Electronic Device.


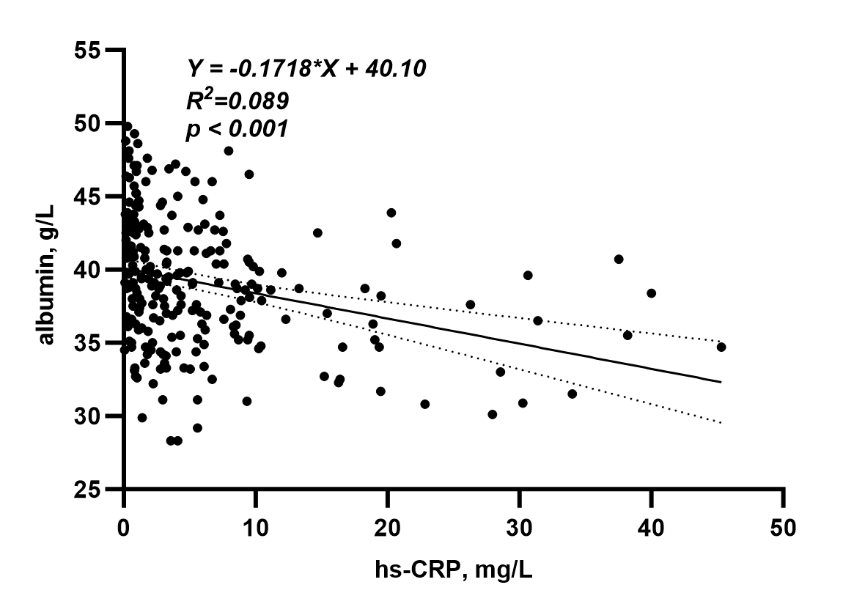


Supplementary Figure 1. Correlation analysis between hs‑CRP and albumin. hs-CRP, high sensitivity C-reactive protein.


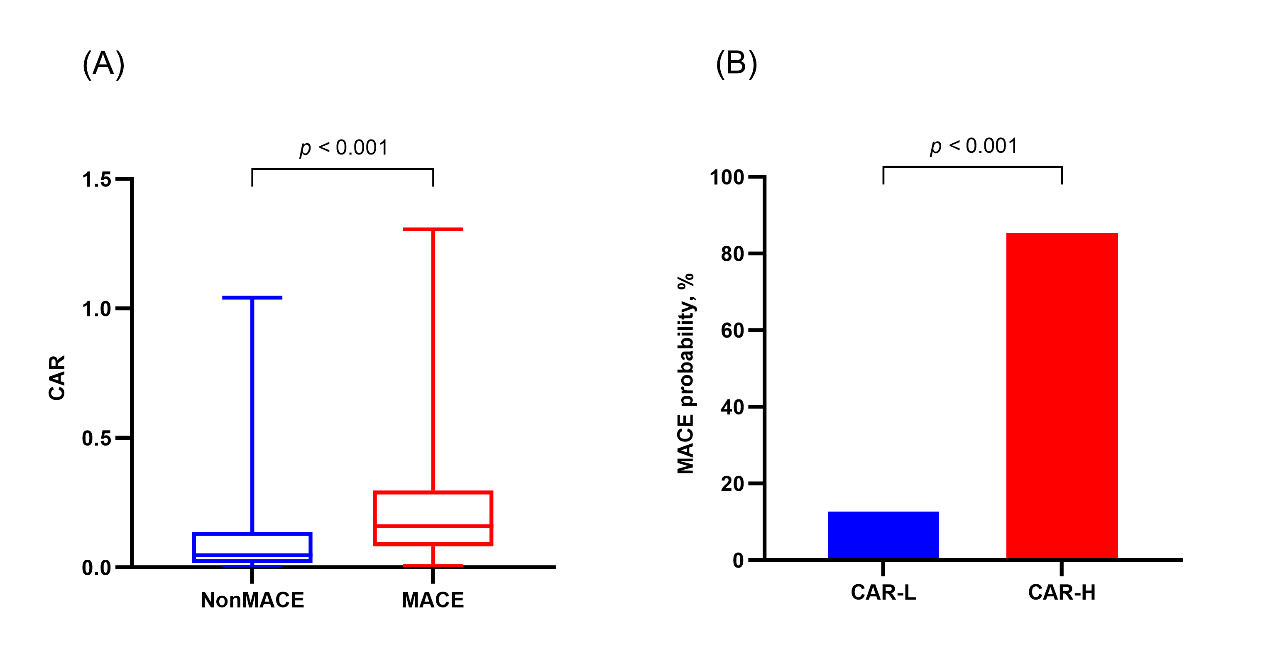


Supplementary Figure 2. Association between CAR and primary outcome. (A) Comparison of CAR levels between non-MACE and MACE groups; (B) Comparison of MACE incidence between different CAR levels. CAR, high-sensitivity C-reactive protein to albumin ratio; MACE, major adverse cardiovascular events.
